# Exploring pericyte and cardiac stem cell secretome unveils new tactics for drug discovery^☆^

**DOI:** 10.1016/j.pharmthera.2016.11.007

**Published:** 2017-03

**Authors:** Georgina M. Ellison-Hughes, Paolo Madeddu

**Affiliations:** a Centre of Human & Aerospace Physiological Sciences, Centre for Stem Cells and Regenerative Medicine, Faculty of Medicine & Life Sciences, Guy's Campus, King's College London, London SE1 1UL, United Kingdom; b Chair Experimental Cardiovascular Medicine, Bristol Heart Institute, School of Clinical Sciences University of Bristol Level 7, Bristol Royal Infirmary, Upper Maudlin Street, Bristol BS2 8HW, United Kingdom

**Keywords:** Abi3bp, ABI Family Member 3 Binding Protein, Ang, Angiopoietin, CSCs, Cardiac stem cells, CDCs, Cardiosphere-derived cells, CM, Conditioned medium, CHD, Coronary heart disease, DPP-4, Dipeptidyl peptidase-4, ESCs, Embryonic stem cells, ECs, ECs, EPCs, Endothelial progenitor cells, bFGF, Fibroblast growth factor, FDA, Food and Drug Administration, GLP1, Glucagon-like peptide-1, EPCs, Endothelial progenitor cells, eNOS, Endothelial nitric oxide synthase, FAECs, Fetal aorta ECs, FOXO1, Forkhead box protein O1, G-CSF, Granulocyte-colony stimulating factor, HF, Heart failure, HGF, Hepatocyte growth factor, IGF-1, Insulin growth factor-1, IL, Interleukin, HGF, Hepatocyte growth factor, HUVECs, Human umbilical vascular ECs, MMPs, Metalloproteinases, MI, Myocardial infarction, MCP-1, Monocyte chemoattractant protein-1, MSCs, Mesenchymal stem cells, NHS, National Health System, NRG-1, Neuregulin 1, PDGFβ, Platelet-derived growth factor beta, sFRP1, Secreted frizzled-related protein 1, SCF, Stem cell factor, SDF-1, Stromal cell-derived factor-1, TGF-β1, Transforming growth factor beta1, TNF-α, Tumor necrosis factor, LC-MS/MS, Tandem Mass Spectrometry Detection, VEGF-A, Vascular growth factor A, VPCs, Vascular progenitor cells, Cardiac stem cells, Pericytes, Secretome, Regenerative medicine, Drug discovery

## Abstract

Ischaemic diseases remain a major cause of morbidity and mortality despite continuous advancements in medical and interventional treatments. Moreover, available drugs reduce symptoms associated with tissue ischaemia, without providing a definitive repair. Cardiovascular regenerative medicine is an expanding field of research that aims to improve the treatment of ischaemic disorders through restorative methods, such as gene therapy, stem cell therapy, and tissue engineering. Stem cell transplantation has salutary effects through direct and indirect actions, the latter being attributable to growth factors and cytokines released by stem cells and influencing the endogenous mechanisms of repair. Autologous stem cell therapies offer less scope for intellectual property coverage and have limited scalability. On the other hand, off-the-shelf cell products and derivatives from the stem cell secretome have a greater potential for large-scale distribution, thus enticing commercial investors and reciprocally producing more significant medical and social benefits. This review focuses on the paracrine properties of cardiac stem cells and pericytes, two stem cell populations that are increasingly attracting the attention of regenerative medicine operators. It is likely that new cardiovascular drugs are introduced in the next future by applying different approaches based on the refinement of the stem cell secretome.

## 1 Introduction

Coronary heart disease (CHD) caused by the narrowing of arteries that feed the heart is the UK's single biggest killer, being responsible for ~ 73,000 deaths each year, an average of 200 people each day. Acute myocardial infarctionl (MI) represents the most harmful form of CHD. Over the last decade, mortality due to CHD has declined in the UK, but more people live with secondary consequences. In fact, most of the current treatments are palliative, i.e. they reduce symptoms associated with heart dysfunction, without providing a definitive repair. Consequently, CHD patients undergo a progressive decline in the pumping function of the heart that ultimately leads to heart failure (HF). Today, post-infarct HF is the leading cause of invalidity, hospitalization and mortality in patients over 65. In 2012–13, the UK National Health System (NHS) expenditure for cardiovascular disease was £7.02billion, 63% of which devoted to secondary care (Bhatnagar, Wickramasinghe, Williams, Rayner, & Townsend, 2015) The NHS analysts have predicted a mismatch between total budget and patient needs of nearly £30 billion by 2020/21. Therefore, efficiency actions to increase quality and reduce expenditure growth are essential for all services, including those for treatment and care of CHD patients. However, efficiency alone may not suffice without the introduction of new technologies having a transformative impact on this unmet clinical field.

### 1.1 The urgent need for new therapies

Current care of CHD comprises pharmacotherapy and revascularisation. However, medical treatment can be ineffective as in the case of refractory angina (which has an estimated prevalence of 1.8 million in the USA and an incidence of 30–50,000/year in Europe). Additionally, a steadily increasing number of patients fall into the category in which revascularization cannot be applied or fails because of restenosis. This is especially true of patients with occlusive pathology extending to the microcirculation and diabetic or elderly patients who have had multiple bypasses and stenting operations. Also, the most important limitation of current treatments is that they do not replace cells irreversibly damaged by ischaemia.

Cardiovascular regenerative medicine is a fast-growing field of research that aims to improve the treatment of CHD through innovative restorative methods, such as gene therapy, stem cell therapy and tissue engineering (Assmus et al., 2002, Wollert et al., 2004). Clinical studies with skeletal myoblasts, bone marrow-derived cells, mesenchymal stem cells (MSCs) and cardiac stem cells (CSCs) have shown feasibility and initial evidence of efficacy (Assmus et al., 2002, de Jong et al., 2014, Hare et al., 2009, Menasche et al., 2008, Sant'anna et al., 2010). After multiple systematic reviews and meta-analyses, the consensus is that transplantation of adult bone marrow cells modestly improves ventricular function, infarct size, and remodeling in patients with CHD compared with standard therapy, and these benefits persist during long-term follow-up (Martin-Rendon, 2016). Bone marrow cell transplantation also reduces the incidence of death, recurrent MI, and stent thrombosis in patients with CHD (Jeevanantham et al., 2012). Moreover, Steven Chamuleau, Andreas Zieher and collegaues have recently utilized interaction models in a multivariable fashion to identify subgroups of patients that are defined as potential treatment responders, while simultaneously correcting for relevant factors that affect general disease outcome. This kind of approach could be the next step towards optimal cell therapy in clinical care (Zwetsloot et al., 2016).

The SCIPIO clinical trial, the first in man to investigate c-kit + CSCs, reported that 16 patients with ischemic cardiomyopathy received intracoronary infusions of 0.5-1 × 10^6^ c-kit +, autologous CSCs and compared to controls these patients benefited from an 8 and 12 unit increase in left ventricular ejection fraction, 4 and 12 months after infusion, respectively (Bolli et al., 2011). A subset of 7 patients was subject to cMRI analysis, which showed that the infarct region had significantly decreased in size by ~ 10 g up to 12 months following c-kit + CSC transplantation (Bolli et al., 2011).

However, there is a persisting dispute regarding the mechanisms underpinning the benefit of cell therapy. The direct contribution of transplanted cells in vascular and cardiac reconstitution has been questioned (Balsam et al., 2004, Murry et al., 2004), and presently the concept of paracrine promotion of spontaneous healing processes prevails (Gnecchi et al., 2005, Tang et al., 2016a, Tang et al., 2016b). Indeed, the general consensus is that cell therapy and resultant improvements in cardiac function and decreased infarct size in human trials is due to a ‘paracrine’ effect (Tang et al., 2016a, Tang et al., 2016b). However, the lack of cardiomyocyte differentiation capability of bone marrow cells or CSCs could be due to lack of characterisation of the transplanted cell type, poor cell survival and retention, hostile host environment and subsequent restriction of cell proliferation, integration and differentiation in this damage-regeneration infarct model.

Despite the adult mammalian heart being composed of terminally differentiated cardiomyocytes that are permanently withdrawn from the cell cycle (Chien and Olson, 2002, Nadal-Ginard, 1978), it is now apparent that the adult heart has the capacity, albeit low, to self-renew cardiomyocytes over the human lifespan (Bergmann et al., 2012, Bergmann et al., 2015). This is supported by the detection of small, newly-formed, immature cardiomyocytes, which incorporate BrdU/EdU and/or stain positive for Ki67, Aurora B, and embryonic/neonatal myosin heavy chain, as well as cardiomyocytes undergoing mitosis, under normal conditions and in response to diverse pathological and physiological stimuli (Bergmann et al., 2015, Bostrom et al., 2010, Ellison et al., 2013, Urbanek et al., 2003, Urbanek et al., 2005, Waring et al., 2014). The source of these newly formed cardiomyocytes is a matter of debate (Laflamme & Murry, 2011). Three main sources of origin of the new cardiomyocytes have been claimed: (a) circulating progenitors, which through the bloodstream home to the myocardium and differentiate into cardiomyocytes (Quaini et al., 2002); (b) mitotic division of the pre-existing cardiomyocytes (Bersell et al., 2009, Bostrom et al., 2010, Engel et al., 2006, Senyo et al., 2013); and (c) a small population of resident multipotent stem cells able to differentiate into the main cell types of the heart (i.e., cardiomyocytes, smooth and endothelial vascular and connective tissue cells) (Rasmussen et al., 2014, Torella et al., 2007).

Blood-borne precursors, although well documented for having a role in inflammation and healing, and when adult mouse bone marrow cells were injected into the chick embryo they converted to a myocardial phenotype (Eisenberg, Burch, & Eisenberg, 2006), their cardiomyogenic potential in the damaged adult heart is very limited, if any (Ellison et al., 2013, Loffredo et al., 2011). The evidence so far presented in support of re-entry of terminally differentiated cardiomyocytes into the cell cycle has been limited to show division of cells that express proteins of the contractile apparatus in their cytoplasm (Bersell et al., 2009, Bostrom et al., 2010, Kuhn et al., 2007, Senyo et al., 2013). This evidence is equally compatible with new myocyte formation from the pool of multipotent cardiac stem/progenitor cells, which as precursor cells express contractile proteins and because newly born myocytes are not yet terminally differentiated they are capable of a few rounds of division before irreversibly withdrawing from the cell cycle (Nadal-Ginard, 1978, Nadal-Ginard et al., 2003). However, the mechanisms underlying a strict postmitotic state in the heart during pathological remodeling have yet to be fully elucidated (Zebrowski, Becker, & Engel, 2016).

The best documented source of the small, immature, newly formed cardiomyocytes in the adult mammalian heart, including the human (Torella, Ellison, Mendez-Ferrer, Ibanez, & Nadal-Ginard, 2006), is a small population of endogenous cardiac stem and progenitor cells (eCSCs) distributed throughout the atria and ventricles, which are clonogenic, self-renewing and can give rise to functional cardiomyocytes and vasculature in vitro and in vivo. Importantly, owing to genetic labelling and transitional tracking it is now documented that newly formed cardiomyocytes observed in the adult mammalian heart are the product of eCSC differentiation (Ellison et al., 2013, Hsieh et al., 2007, van Berlo and Molkentin, 2014).

Here, we provide an overview of current knowledge regarding the therapeutic potential of using the stem cell-derived secretome instead of source stem cell therapy to repair and regenerate the damaged heart. Furthermore, we illustrate methodological aspects of secretome-based cardiovascular regenerative medicine, with particular reference to functional transcriptomics and proteomics as a combinatory strategy to cherry pick the most beneficial components of the stem cell secretome. Finally, we report current evidence regarding the salutary aspects of the secretome from two stem cell populations, namely CSCs and pericytes.

## 2 Generation of a therapeutic product from stem cell secretome

Stem cells secrete potent combinations of cytokines, growth factors, enzymes, microvesicles/exosomes and genetic material, which help cardiac repair and regeneration at multiple points. The stem cell secretome supports cardiomyocyte survival and proliferation, differentiation of resident stem cells, and neovascularization, while limiting inflammatory and pro-fibrotic processes (Baraniak and McDevitt, 2010, Gnecchi et al., 2005, Rao et al., 2015, Zhou et al., 2011). Some paracrine factors have pleiotropic actions. For instance, one of the key pathways in stem cell-based cardiac repair is the stromal cell-derived factor-1 (SDF-1)/CXCR4 axis. It was proposed that substituting SDF-1 gene therapy for source stem cells might represent a sensible therapeutic approach (Penn, 2009). The blinded placebo-controlled STOP-HF trial demonstrated a single endocardial administration of plasmid SDF-1 is safe, attenuates left ventricle remodeling and improves ejection fraction in ischaemic cardiomyopathy (Chung et al., 2015). However, there are also paracrine factors that boost specific responses, thereby providing a more defined approach for selective treatments inspired by the stem cell secretome. For instance, mesenchymal stem cell-derived Insulin Growth Factor-1 (IGF-1) and ABI Family Member 3 Binding Protein (Abi3bp) reportedly induce resident CSC mobilization (Mourkioti & Rosenthal, 2005) and differentiation to the cardiac lineage, (Engels et al., 2014, Hodgkinson et al., 2014) while CSC-derived basic Fibroblast Growth Factor (bFGF), Vascular Growth Factor-A (VEGF-A) and Hepatocyte Growth Factor (HGF) exert potent pro-angiogenic effects (Rao et al., 2015, Zhou et al., 2011).

Intracoronary administration of IGF-1 and HGF, in doses ranging from 0.5 to 2 μg HGF and 2 to 8 μg IGF-1, just below the site of left anterior descendent occlusion, 30 min after MI during coronary reperfusion in the pig, triggered a regenerative response from the CSCs, which is potent and able to produce physiologically significant regeneration of the damaged myocardium (Ellison et al., 2011). IGF-1 and HGF induced CSC migration, proliferation and functional cardiomyogenic and microvasculature differentiation. Furthermore, IGF-1/HGF, in a dose-dependent manner, improved cardiomyocyte survival, and reduced fibrosis and cardiomyocyte reactive hypertrophy. Interestingly, the effects of a single administration of IGF-1/HGF is still measurable 2 months after its application, suggesting the existence of a feedback loop triggered by the external stimuli that activate the production of growth and survival factors by the targeted cells, which explains the persistence and long duration of the regenerative myocardial response. These histological changes were correlated with a reduced infarct size and an improved ventricular segmental contractility and ejection fraction at the end of the follow-up assessed by cMRI (Ellison et al., 2011).

Neuregulin-1 (NRG-1) is another key factor implicated in stimulating cardiac repair and regeneration (Wadugu and Kuhn, 2012, Waring et al., 2014). An Ig-domain containing form of NRG-1β, also known as glial growth factor 2 (GG2) has been shown to improve left ventricular ejection fraction and remodeling in pigs post-MI, compared to controls (Galindo et al., 2014). It is thought that NRG-1 imparts functional benefits by activating and increasing eCSC proliferation (Waring et al., 2014), inducing cardiomyocyte replacement (Bersell et al., 2009, Cohen et al., 2014, Polizzotti et al., 2015). A bioengineered hydrogel system enables targeted and sustained intramyocardial delivery of NRG-1, activating the cardiomyocyte cell cycle and enhancing ventricular function in a murine model of ischemic cardiomyopathy (Cohen et al., 2014), protecting cardiomyocytes from apoptosis and improving mitochondrial function (Galindo et al., 2014). However, the role of NRG-1 in inducing cardiomyocyte proliferation in the adult heart has been challenged, with NRG1β1 treatment not increasing cardiomyocyte DNA synthesis and consequent cardiomyocyte renewal in normal or infarcted adult mouse hearts (Reuter, Soonpaa, Firulli, Chang, & Field, 2014). Therefore, the role of NRG-1 administration in inducing cardiomyocyte proliferation and replacement in the adult failing heart remains controversial.

The stem cell secretome is collected in a form of cell culture conditioned medium (CM) or supernatant. The use of stem cell-derived secretome has several advantages compared to the use of stem cells, as CM can be manufactured, sterilized, freeze-dried, packaged, stored and transported more easily. Therefore, the stem cell-derived secretome has a promising prospect to become a successful pharmaceutical/medicinal product for regenerative medicine. On the other hand, variability represents a major limitation. The first level of inconsistency is represented by inter-individual variability, due to the patient's characteristics. Culture conditions, growth media and passages also affect the composition of CM, as recently reviewed (Pawitan, 2014). In some occasions, changes in culture conditions are intentionally introduced to enhance the production of certain biological components. For instance, stem cells could be exposed to hypoxic conditions, with the aim of stimulating the production of growth factors (Di Santo et al., 2009, Ranganath et al., 2012). The three-dimensional spheroid culture of human adipose-derived stem cells with clinically relevant medium composed of amino acids, vitamins, glucose, and human serum leads to 23- to 27-fold higher production of angiogenic factors than that by conventional monolayer culture (Bhang et al., 2014). On the other hand, the purity of preparations may become an issue when complete growth medium is used, or collection of CM is performed shortly after removal of growth medium supplements. In this case, contamination may derive from the presence of left-over serum carrier proteins, due to dynamic recycling of extracellular proteins by cellular vesicles (Iso et al., 2014). The presence of serum or culture media in the CM- or supernatant-derived product might elicit immune responses and/or side effects. In this respect, alternative methods to culture cells (such as the adoption of serum-free culture, etc) and the refinement of the CM-derived secretome products should be considered. However, a recent study indicates that the formation of complexes of serum-derived immunoglobulins with paracrine factors could enhance the stability and biological activity of the CM therapeutic component (Rao et al., 2015). A challenging objective of future research is to define the whole composition of crude CM preparations, measure activity of the main components and determine therapeutic doses. In this respect, manufacturing and quality control protocols need to be further refined and standardized to accomplish the classical regulatory path for drugs. The goal is to generate synthetic versions, inspired by the stem cell secretome, containing consistent dosages of therapeutic factors for the treatment of thousand patients.

### 2.1 Methodologies to determine the therapeutic component

Different methods have been used for studying the secretome composition and for distinguishing the essential components that are advantageous for therapeutic applications. Multiplex antibody-based techniques, such as antibody arrays, have high sensitivity (in the range of 1–10 pg/ml), specificity, and reproducibility across a broad range of concentrations. High-throughput analysis of the human MSC secretome using a human cytokine antibody array has identified about 40 proteins with high expression levels (Parekkadan et al., 2007). Studies using multiplex antibody-based arrays showed the contribution of MSC-derived paracrine factors in determining cardiac improvement in a swine MI models (Nguyen et al., 2010) and the participation of angiogenin, a secreted ribonuclease that inhibits protein translation under stress conditions, in promotion of cardiomyocyte survival by intramyocardially injected bone marrow-derived mononuclear cells (Ascione et al., 2015). However, a common drawback of antibody-based techniques (e.g., ELISA and antibody arrays) consists of the limited availability of antibodies to detect secreted proteins.

Liquid Chromatography with Tandem Mass Spectrometry Detection (LC-MS/MS) has been widely used for characterizing the secretome profile. For example, a study using this technique demonstrated that preconditioning of human adipose-derived MSCs with Tumor Necrosis Factor-alpha (TNF-α) leads to enhanced expression of cytokines and chemokines such as Interleukin-6 (IL-6), Interleukin-8 (IL-8), Monocyte chemoattractant protein-1 (MCP-1), metalloproteinases (MMPs), Pentraxin-related protein, and Cathepsin L. in CM (Lee et al., 2010). On the other hand, gel-based and LC-MS/MS techniques have limited sensitivity to detect molecules that are present in fmol concentrations (Ranganath et al., 2012).

**Fig. 1:**
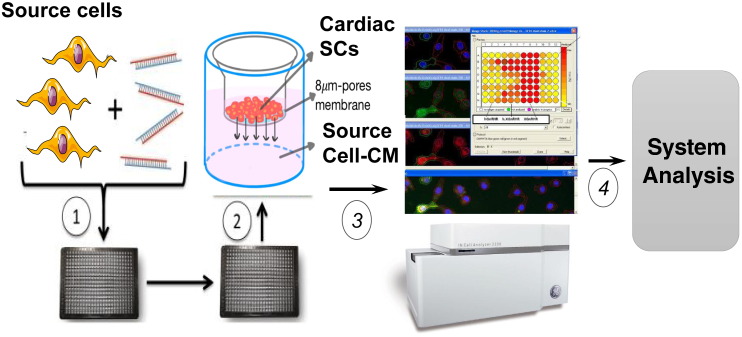
Cardiac stem cell migration-based siRNA secretome screening. (1) Source cells are transfected with pools of multiple siRNAs against single mRNA targets from a list of pro-migratory genes. (2) After removal of transfecting agents, source cells are cultured for 48 h and then the CM is collected and transferred to a multi-well plate assay for assessment of cardiac stem cell migration. (3) Migrated stem cells (stained with nuclear Hoechst) are enumerated using a high content Cell Analyzer. (4) Data could be integrated with results from other platforms, such as Tandem Mass Spectrometry, to perform system analysis of the secretome.

RNA interference, by siRNA or shRNA, represents a powerful method to evaluate the function of candidate genes (Lemons, Maurya, Subramaniam, & Mercola, 2013). Throughput interference screening of mRNAs and microRNAs in cell-based assays can also help to decipher the functional importance of single components of the stem cells secretome. It is necessary to validate the outcome of silencing by rescue experiments where the CM from silenced cells is supplemented with a candidate recombinant protein to determine if it restores the original phenotype. Fig. 1 illustrates an application of siRNA technology to the discovery of CM components implicated in the activation of stem cell migration.

A more systematic approach to tackling the secretome complexity combines LC-MS/MS detection, antibody arrays, microarrays, and bioinformatics. This approach identified 201 unique proteins (132 using LC-MS/MS and 72 using antibody arrays) typical of human MSC secretome (Sze et al., 2007). Also, Sze exploited a computational analysis of data to predict the roles of the secretome components in metabolism, immune response, and development.

### 2.2 Pre-clinical cardiovascular studies using stem cell-derived secretome

Some preclinical studies have demonstrated that delivery of stem cell-derived CM has salutary effects in models of limb and myocardial ischaemia. Table 1 summarizes some fundamental investigations that evaluated the effect of crude CM preparations. A study using an intra-muscular injection of endothelial progenitor cell-derived CM (EPC-CM) collected under hypoxia showed outcomes (i.e. tissue revascularization and functional recovery) similar to cell transplantation in a model of limb ischaemia (Di Santo et al., 2009). Similar benefits have been reported with the use of CM from human amniotic fluid stem cells (Mirabella, Cilli, Carlone, Cancedda, & Gentili, 2012), cord blood-derived EPCs (Kim et al., 2010) and fetal aorta CD133 vascular cells (Barcelos et al., 2009) in limb ischaemia and wound healing models and with CM from EPCs (Hynes et al., 2013) and MSCs in MI (See et al., 2011, Timmers et al., 2007, Timmers et al., 2011, Yang et al., 2013). Furthermore, the CM of human embryonic stem cell-derived ECs reportedly restored the healing activity of circulating proangiogenic cells from diabetic patients upon combined injection in ischaemic limbs of severe combined immunodeficient mice (Ho et al., 2012). One important limitation of using CM for therapeutic uses consists of the rapid degradation of therapeutic components in vivo. To obviate this drawback, some investigators have used repeated injections of the CM in preclinical studies, but this approach may be unpractical for clinical applications. We have shown that perivascular transplantation of micro-incapsulated glucagon like peptide-1 (GLP1)-expressing stem cells enables long-lasting cell retention in the ischaemic site as well as prolonged paracrine activity, resulting in robust limb arteriogenesis (Katare et al., 2013). Results of cytometric bead arrays on tissue collected 1 week after implantation showed the expression of human angiogenic proteins in ischaemic muscles, indicating the persistence of paracrinally active human cells.

**Table 1:** Cardiovascular preclinical trials using stem cell-derived secretome.

Disease/delivery	Animal model	Source of CM	Outcome	Reference
Limb ischaemia— Daily injection of 40 μl of human adipose-derived stem cell (ADSC) CM for 7 days into the gracilis muscle	Athymic mice	Human adipose-derived stem cells	Enhanced endothelial cell growth, CD34^+^ cell mobilization from bone marrow, and bone marrow cell homing to the ischemic region, resulting in improved blood vessel density, limb salvage, and blood perfusion.	Bhang et al. (2014)
Limb ischaemia— Single injection of human embryonic stem cell-derived endothelial-like cell (ESC-EC) CM and/or circulating proangiogenic cells (PACs) into the gracilis muscle	SCID mice	Human embryonic stem cell-derived endothelial-like cells	Neither diabetic PACs nor CM from ESC-ECs improve blood flow recovery and angiogenesis. In contrast, both transplantations of proangiogenic cells from controls or diabetic patients supplemented with ESC-ECs CM improve blood flow and angiogenesis.	Ho et al. (2012)
Limb ischaemia— Three weekly intramuscular injections of endothelial progenitor cells (EPCs), EPC-CM, or control medium	Athymic nude rats	Human peripheral blood endothelial progenitor cells	Both EPC-CM and EPCs increase limb blood flow assessed and neovascularization. EPC-CM transplantation stimulates the mobilization and recruitment of bone marrow-derived EPCs.	Di Santo et al. (2009)
Limb ischaemia— Two intramuscular weekly injections of human amniotic liquid derived stem cells (AFSC) CM (topically applied to thigh muscles) for a total treatment-duration of two weeks	SCID mice	Human amniotic liquid derived cKit stem cells	Increased arteriogenesis, capillary density, total perfusion area, and mobility.	Mirabella et al. (2012)
Myocardial infarction— Peri-infarct injection of human adipose-derived stem cells (ADSC), ADSC-CM or control medium immediately after MI	SCID and C57BL/6 mice	Human adipose-derived stem cells	Improved cardiac function, reduced infarct size, increased reparative angiogenesis, reduced cardiomyocyte apoptosis The effect of ADSCs on the first 3 outcomes was superior to that of ADSC-CM.	Yang et al. (2013)
Myocardial infarction— Intramyocardial injections of either concentrated CM derived from STRO-3- mesenchymal precursor cells cultured in serum-free medium or control medium at 48 h after MI	Athymic nude rats	Human STRO-3- mesenchymal precursor cells	Improved ventricular function, reduced ventricular dilatation, and infarct size, increased neovascularization.	See et al. (2011)
Myocardial infarction— Intravenous treatment with CM from human embryonic stem cells-derived MSCs or control medium initiated 4 h after coronary artery ligation with the treatment continued for 7 days twice daily via a catheter inserted into the jugular vein	Pigs	Human embryonic stem cells-derived MSCs	Increased capillary density, reduced infarct size improved myocardial performance.	Timmers et al. (2011)
Myocardial infarction— At the end of 2 h reperfusion, three cycles of intracoronary infusion CM from porcine endothelial progenitor cells or vehicle	Pigs	Porcine peripheral blood endothelial progenitor cells	Increased angiogenesis, improved cardiomyocyte remodeling and contractility.	Hynes et al. (2013)

Growing evidence suggests stem cells used in regenerative medicine secrete some form of extracellular vesicles, including microvesicles and exosomes. Exosomes represent a relatively homogeneous subset of secreted membrane vesicles that are formed by direct budding of the plasma membrane into early endosomes (Sahoo & Losordo, 2014). Exosomes derived from stem cells activate angiogenesis and cytoprotection and modulate inflammation and apoptosis (Lee et al., 2012, Mackie et al., 2012, Sahoo et al., 2011). Seminal preclinical studies from Sahoo et al. have shown that exosomes may represent a significant component of the paracrine effect of stem cell transplantation for therapeutic angiogenesis (Sahoo et al., 2011). Important insights are also emerging from mechanistic studies. Exosomes derived from various stem cells enhance the myocardial viability and prevent adverse heart remodeling through a reduction in oxidative stress and Akt activation (Arslan et al., 2013). Furthermore, exciting new research suggests that exosomes directly communicate with the target cells by delivering specific microRNAs and other small RNAs characteristic of their parental cell of origin (Mackie et al., 2012, Yuan et al., 2009). Analysis of microRNAs expression in embryonic stem cell-derived exosomes revealed high expression of embryonic-specific microRNAs belonging to the miR-294 family. This microRNA regulates fundamental properties of embryonic stem cells (ESCs), including pluripotency, cell cycle, and proliferation and participates in the beneficial effect of ESC-derived exosomes transplantation in the injured heart (Khan et al., 2015). A recent study showed the pathogenic communication between vascular ECs and pericytes in the diabetic microvasculature is mediated by the shedding of endothelial microparticles carrying miR-503, which transfer miR-503 from ECs to vascular pericytes (Caporali et al., 2015).

To date, no clinical trial employing stem cell-derived CM for the treatment of cardiovascular disease has been reported. Two pilot studies described potential therapeutic activity of MSC CM for hair follicle regeneration and resurfacing wound healing in humans (Fukuoka and Suga, 2015, Shin et al., 2015, Zhou et al., 2013). These initial trials are expected to encourage clinical studies for the treatment of cardiovascular patients.

### 2.3 Commercialization of the stem cell secretome

The stem cell secretome is receiving increasing attention not only from researchers but also from industry. Recently, US-based stem cell therapeutic company Stemedica announced worldwide manufacturing license of VitriLife to their subsidiary StemProtein (Source: Stemedica Cell Technologies, Inc.). The patent covers a novel vaporization technology that immobilizes biologicals in glassy carbohydrates (a VitriLife™ state) thus allowing storage at room temperature for several years. The company claims VitriLife-preserved proteins are superior in activity compared to lyophilized proteins. The thermostable mixture from VitriLife processed stem cell-derived CM contains therapeutic biologicals, such as cytokines, chemokines, growth factors, proteins, mRNAs, and microRNAs. The new technology may provide an efficient way for converting stem cell-derived factors into powders that retain natural efficacy for eventual bulk processing and packaging of product and delivery to medical markets. Stemedica has plans to commercialize the product for many disease conditions, from skin problems to Alzheimer's disease, but has not released yet any information about regulatory status.

Stem cell-derived CM in cosmetic products has been commercialized and sold world-wide. Examples include Regenica cream made with Human Fibroblast Serum Free CM by SkinMedica, *anti*-aging non-embryonic stem cell extracts manufactured by Lifeline Skin Care, stabilized CM from placenta- and umbilical cord-derived human stem cells from Blue Horizon, and serum-free adipose-derived MSC CM from Celprogen.

The regulatory process of CM-derived products remains vague. Despite the fact stem cell-derived cosmetic products have been commercialized for a decade as components of skin creams, only recently Food and Drug Administration (FDA) has pronounced a statement that these compounds should be considered drugs under the Federal Food, Drug, and Cosmetic Act (http://www.ipscell.com/2016/08/3-more-fda-warning-letters-to-stem-cell-cosmetics-makers/). Therefore, marketing of these products with claims evidencing the use in the diagnosis, cure, mitigation, treatment, or prevention of disease violates the Act. If this applies to skin creams, even more stringent regulation should be installed for the therapeutic use of CM in human cardiovascular diseases.

## 3 Exploiting the secretome of CSCs and pericytes for cardiovascular repair

One important caveat of secretome-based therapy is represented by the specificity of the source cell about the target tissue. It is, in fact, more likely that a therapeutic effect is achieved by exploiting the paracrine activity of tissue-specific cells rather than using cells isolated from a different tissue. In this respect, CSCs and pericytes may be uniquely suited to produce paracrine factors instrumental to cardiac and vascular repair and regeneration.

The first report of CSCs able to repopulate the adult heart was in 2003 (Beltrami et al., 2003). Following this initial report, several cardiac stem/progenitor cell types have been identified based on the specification of membrane markers and transcription factors and resident CSCs exhibit spontaneous regenerative capacity when tested in an appropriate injury model (Ellison et al., 2013). Table 2 provides a summary of different CSC subtypes based on antigenic characteristics.

**Table 2:** Cardiac stem cells subtypes based on antigenic characteristics.

Subtype	cKit	CD34	Sca-1	Abcg2	Wt1	CD105	GATA4	MEF2C	NKX2–5	CD166	CD45	PDGFRα	CD90	FLK1
c-Kit CSCs	+	−	+	+	−	+	+	+	±	+	−	+	±	±
Sca-1 CSCs	low	−	+		±		+	+	low		−	+		−
Side population	+	+	+	+	±		−		−		+	+		
Cardiosphere-derived cells	low	+	+	+		+					+			
Cardiac resident colony-forming unit-fibroblast	low		+		+	+					−	+	+	−
Isl-1pos cardiac progenitor cells	−		−				+		+					
Epicardial stem cells	−		+		+									

C-kit + CSCs (Beltrami et al., 2003, Ellison et al., 2013) Sca1pos CSCs (Oh et al., 2003); side population (SP) cells) (Martin et al., 2004); Cardiosphere-derived cells (Chimenti et al., 2012); cardiac resident colony-forming unit-fibroblast (cCFU-Fs) (Chong et al., 2011); and the Isl-1pos cardiac progenitor cells (Laugwitz, Moretti, Caron, Nakano, & Chien, 2008); Epicardial stem cells (Smart et al., 2011).

Pericytes are mural cells that encircle capillaries in different organs. Pericyte-like cells that express stem cell markers and possess clonogenic expansion capacity have been identified in the adventitia of large vessels from human fetuses (Invernici et al., 2007) and adult individuals (Campagnolo et al., 2010). They share several markers that are typical of microvascular pericytes but do not express the surface marker CD146 (Table 3). Preclinical studies have shown that transplantation of microvascular pericytes and adventitial pericyte-like stem cells exert therapeutic actions in models of myocardial (Avolio et al., 2015b, Chen et al., 2013, Katare et al., 2011) and peripheral ischaemia (Campagnolo et al., 2010, Dar et al., 2012, Gubernator et al., 2015). In the infarcted mouse heart, combined transplantation of human adventitial pericyte-like stem cells and CSCs, reduced the infarct size and improved indexes of contractility by the promotion of angiogenesis, inhibition of cardiomyocyte apoptosis, and attraction of resident and circulating reparative cells (Avolio, Meloni, et al., 2015).

**Table 3:** Pericyte subtypes based on antigenic characteristics.

Subtype	CD146	NG2	PDGFRβ	PDGFRα	CD34	CD45	CD73	CD44	CD105	CD45	cKit	CD90	CD26	CD31
Microvascular pericytes	+	+	+		−	−				−				
CD146-pos cardiac pericytes	+	+	+	+	−	−	+	+	+	−	−			−
CD146-neg cardiac pericytes	−	+	+		+*		−	+	+	−		low		−
Adventitial pericyte-like stem cells	−	+	+		+*	−	+	+	+	−		+	+	−

Microvascular pericytes (Corselli et al., 2013, Dellavalle et al., 2011); CD146-pos cardiac pericytes (Chen et al., 2015); CD146-neg cardiac pericytes (Avolio, Rodriguez-Arabaolaza, et al., 2015); Adventitial pericyte-like stem cells (Campagnolo et al., 2010, Chen et al., 2012, Klein et al., 2011). *CD34 is used for immunomagnetic sorting of adventitial pericyte-like stem cells but the antigen is not expressed after culture expansion of the isolated cells.

The ultimate goal for heart repair and regeneration is to restore muscle mass, and new cardiomyocyte formation following cell transplantation or ‘paracrine’ cardiac regenerative growth factor overexpression or administration has been demonstrated in different animal models of MI (Ellison et al., 2011, Hatzistergos et al., 2010, Koudstaal et al., 2014, Limana et al., 2007, Loffredo et al., 2011, Madonna and De Caterina, 2011, Martin-Rendon, 2016, Nadal-Ginard, 1978, Nadal-Ginard et al., 2003, Tang et al., 2016a, Tang et al., 2016b, Wei et al., 2015). Whether these newly formed cardiomyocytes derive from re-entry of the cell cycle of pre-existing cardiomyocytes, or more likely from the resident eCSC population (Loffredo et al., 2011), has yet to be determined. Moreover, there are a number of technical limitations to prove myogenesis in these models, and the reader is directed to comprehensive reviews on this subject (Zebrowski et al., 2016).

### 3.1 Evidence for paracrine healing properties of pericytes

In 2007, Invernici et al. (2007) reported the identification of a population of pericyte-like cells in the human fetal aorta composed of undifferentiated mesenchymal cells that coexpress endothelial and myogenic markers. Following supplementation of the culture medium with VEGF-A or Platelet-derived Growth Factor beta (PDGFβ), these cells gave rise to a mixed population of mature endothelial and mural cells. In consideration of the vascular lineage commitment, Invernici named fetal aorta-derived cells with the term *vascular progenitor cell*s (VPCs). Undifferentiated VPCs produce large amounts of immunoreactive VEGF-A and Angiopoietin-2 (Ang-2). Interestingly, the paracrine pattern was profoundly altered on serum-induced VPC differentiation. In fact, in the differentiated cell CM, the VEGF-A amount was negligible, whereas Ang-2 and SDF-1 reached 3.5- and 7.0-fold higher levels than in the CM of undifferentiated cells. These results indicate that VPCs produce angiogenic factors and that the concentration of these factors in the CM varies according to cell maturation. The angiogenic properties of human VPC CM were confirmed in an in vitro proliferation assay with human endothelial cells (ECs). The CM from VPCs stimulated the growth of both human umbilical vascular ECs (HUVECs) and fetal aorta ECs (FAECs), whereas CM produced by VPCs on differentiation was ineffective. The complex angiogenic composition of VPC-derived CM was confirmed because its proangiogenic effect was reduced, but not abolished, by the addition of *anti*-VEGF-A antibodies. In a subsequent study investigating the therapeutic activity of human VPCs in ischemic wound healing, Barcelos et al. (2009) used a cytokine bead array to identify additional proangiogenic factors. They found that undifferentiated VPCs secrete high levels of IL-6 and IL-8, VEGF-A, granulocyte-colony stimulating factor (G-CSF) and MCP-1. Administration of the CM from undifferentiated VPCs instead of cells supported wound closure and neovascularization in mice, whereas the CM of differentiated VPCs was ineffective. Furthermore, neutralizing antibodies against VEGF-A or IL-8 inhibited the healing effect of VPC-derived CM, thus confirming the key role of both factors in wound closure and capillarization, whereas capturing IL-6 was ineffective. Importantly, the study delineated the signaling pathway activated by the VPC secretome in target cells. In fact, VPC-derived CM induced the phosphorylation of Akt at Ser473 as well as of endothelial nitric oxide synthase (eNOS) and Forkhead box protein O1 (FOXO1) in HUVECs. Also, transfection of HUVECs with dominant-negative Akt remarkably reduced the pro-survival effect of CM. In consideration of the link between the VEGF-A and Wnt pathways, Barcelos further explored the involvement of Wnt in the promotion of angiogenesis by VPCs. They found that the Wnt antagonist Secreted frizzled-related protein 1 (sFRP1), but not Dkk-1 an inhibitor of the canonical Wnt signaling pathway, abolishes the facilitation of wound closure and reparative angiogenesis by VPC-derived CM. The editorial comment to Barcelos' study (Chen et al., 2014) highlights this was the first to demonstrate the efficacy and associated healing mechanisms of topical therapy with human progenitor cell-derived CM in a preclinical model of diabetic ischemic foot ulcer. Adding a level of complexity to this system, miR-15a and miR-16-1 can antagonize Wnt signaling (Bonci et al., 2008). Importantly, the two microRNAs impair the biological functions of proangiogenic cells, and their expression is increased in the proangiogenic cells and serum of patients with critical limb ischaemia (Spinetti et al., 2013).

**Fig. 2:**
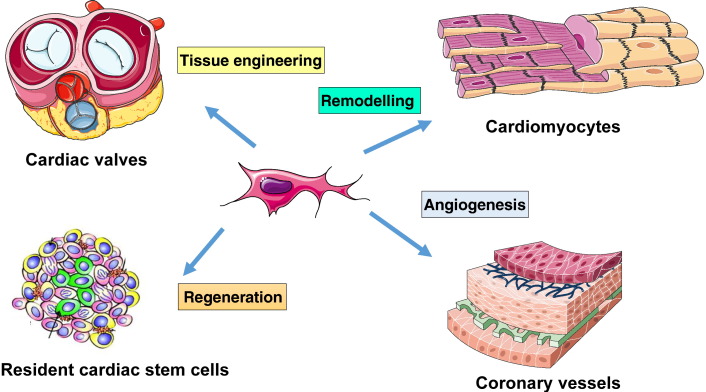
Paracrine action of pericytes. Isolated from small surgical leftovers of vascular or cardiac tissue, pericytes are transplanted in the heart or used for valvular tissue engineering. They release paracrine factors that (1) improve cardiomyocyte survival and inhibit fibrosis, therefore preventing adverse remodeling; (2) exert proangiogenic activity; and (3) promote recruitment of endogenous stem cells. In the case of tissue engineering, repopulation of valvular grafts with pericytes may help the renewal of extracellular matrix protein s through production and release of collagen.

Pericyte-like cells isolated and expanded from the adult saphenous vein produce large amounts of angiogenic factors, in particular, VEGF-A, VEGF-B, Ang-1, and miR-132, which are delivered to neighboring ECs through the establishment of integrin-mediated interactions (Campagnolo et al., 2010, Katare et al., 2011). Secretion of VEGF-A, Ang-1 and miR-132 is further augmented by hypoxia/starvation, which mimics in vitro the environment encountered by cells upon transplantation into ischaemic tissues (Katare et al., 2011). Importantly, anti-miR-132-transfected pericytes were inferior to naïve pericytes or scrambled-transfected pericytes in improving reparative angiogenesis in the infarcted mouse heart. Nonetheless, the improvement afforded by pericytes was not completely abrogated by the miR-132 silencing, thus suggesting the participation of miR-132-dependent and independent mechanisms (Katare et al., 2011). Fig. 2 summarizes the spectrum of the in vivo paracrine interactions between transplanted pericytes and resident cardiomyocytes, CSCs, and ECs.

It has been acknowledged that direct contact and paracrine signaling between different stem cell populations can induce functional changes and influence the susceptibility to a variety of stressors. Also, competitive interaction between stem cells within their native niches reportedly results in survival of the fittest stem cells and death of the most susceptible cells (Leri & Anversa, 2013). We further elaborated this intriguing concept by assessing the reciprocal cross-talk between saphenous vein-derived pericytes and c-Kit CSCs (Avolio, Meloni, et al., 2015). Intra-myocardial injection of adventitial pericytes or CSCs, as a single or combined therapy, increases the abundance of primitive c-Kit + cells in the peri-infarct zone. Also, the CM of the 2 populations exerts a potent chemoattractant activity on murine CSCs, without influencing their proliferation or viability. The two cell populations release similar levels of Hepatocyte Growth Factor (HGF) and stem cell factor (SCF), with no substantial changes in normoxia versus hypoxia or monoculture versus coculture. On the other hand, pericytes secrete the proangiogenic factors Ang-1 and Ang-2 at higher concentrations than CSCs, and a similar trend was observed with bFGF. Interestingly, the secretion of angiopoietins and bFGF by cells in coculture was lower than the average of the 2 cell preparations, thus indicating a negative reciprocal interference. Moreover, hypoxia induced an increase in the secretion of VEGF-A by CSCs, but this effect was not appreeeciated in the coculture. Concerning miR-132 secretion, hypoxia increased its level in pericyte CM, while reducing it CSC CM. An additive effect of the cell coculture was instead noted in the secretion of SDF-1α both under normoxia and hypoxia. Altogether, these data indicate a multifaceted interactive behavior at the level of secretome, which may result in attenuated secretion of VEGF-A, Ang-1, Ang-2, bFGF, and miR-132 in comparison with the prominent cell producer, but the synergic release of SDF-1. To determine whether these phenomena are transcriptionally modulated, we performed quantitative polymerase chain reaction analyses of angiopoietins and SDF-1 mRNA levels in pericytes and CSCs, before and after exposure to each other's CM. Results indicate CM reduces the expression of Ang-2 in both the cell types. Also, CSCs exposed to pericyte CM showed reduced SDF-1 mRNA levels as compared with CSCs exposed to the unconditioned medium. These data suggest a transcriptional interference on Ang-2 and SDF-1. However, while Ang-2 expression was reduced at mRNA and protein level, the increase in SDF-1 content in coculture media was not attributed to the induction of gene transcription, but rather to an increase in secretion rate. Exploring a possible involvement of Dipeptidyl peptidase-4 (DPP-4) we found higher expression levels by CSCs. Furthermore, DPP-4 is downregulated at mRNA and protein level in CSCs exposed to pericytes CM. These findings may have important implication for association cell therapy, where the outcome may depend on the balance between cooperative and competitive interactions at the level of the secretome. For instance, in our study, the combined transplantation of pericytes and CSCs additively reduced the infarct size and promoted vascular proliferation and arteriogenesis, but did not surpass single therapies concerning contractility indexes (Avolio, Meloni, et al., 2015).

Skeletal muscle-derived stem cells have been among the first cell therapy models for regenerative treatment of the infarcted heart (Menasche, 2008). A recent study investigated the therapeutic potential of human skeletal muscle pericytes for treating ischaemic heart disease and mediating associated repair mechanisms in mice (Chen et al., 2013). The authors found that pericyte transplantation attenuates left ventricular dilatation and myocardial fibrosis and improves cardiac contractility in infarcted mouse hearts. In line with findings in saphenous vein-derived pericytes, hypoxia-induced the expression of VEGF-A as well as PDGF-β, Transforming Growth Factor beta1 (TGF-β1), and corresponding receptors while expression of bFGF, HGF, and Ang-1 was repressed.

### 3.2 Resident cardiac pericytes: are they better suited for the heart?

New knowledge on microvessel-associated regenerative precursor cells in cardiac muscle opens prospectives for organ-specific treatments of patients with congenital and acquired heart defects. Peault's team showed that microvascular pericytes within the human myocardium exhibit phenotypes and multipotency similar to their anatomically and developmentally distinct counterparts (Chen et al., 2015). However, they have no ability for skeletal myogenesis, diverging in this respect from pericytes of all other origins. In contrast, a cardiomyogenic potential was evidenced both in vitro and after intra-myocardial transplantation in vivo.

Using the same standard operating protocol employed for the derivation of pericytes from adult saphenous veins, we have isolated and expanded pericyte-like progenitors from small biopsies of congenitally defective hearts (antigenic features illustrated in Table 3) (Avolio, Rodriguez-Arabaolaza, et al., 2015). The long-term objective of research on neonatal cardiac pericytes is their perusal for definitive correction of congenital heart defects (Avolio, Caputo, & Madeddu, 2015). A spectrum of prostheses in the form of conduits, patches, and valves is employed in congenital cardiac surgery, but none of them is perfect. In particular, currently, available grafts do not possess growing capacity and become incompetent with time, thus requiring surgical replacement. The basic concept of tissue engineering is to create living material made by cellularized grafts that, once implanted into the heart, grows and remodels in parallel with the recipient organ. Neonatal pericytes produce a powerful secretome uniquely fit for the purpose. This includes growth factors that promote vasculogenesis and cardiomyogenesis and chemokines able to attract ECs and endothelial progenitor cells instrumental to graft endothelialisation. Comparison with saphenous vein–derived pericytes indicates similarity of the 2 cell populations, though cardiac pericytes secrete more HGF (6-fold), Ang-2 (8-fold), bFGF (4-fold), and VEGF-A (6-fold) than saphenous vein–derived pericytes. Furthermore, neonatal cardiac pericytes release procollagen type 1, a major constituent of the cardiac extracellular matrix, which is fundamental to the maintenance of graft integrity.

The umbilical cord represents a valuable source of stem cells immediately available at birth for corrective strategies. In a recent study, human cord-derived pericytes or cord blood-derived MSCs were delivered before or after alveolar injury into the airways of newborn rats exposed to hyperoxia, a model of bronchopulmonary dysplasia which complicates extreme prematurity and currently lacks efficient treatment (Pierro et al., 2013). Rat pups exposed to hyperoxia showed typical alteration in alveolar growth with enlargement and loss of lung capillaries. Both human cord-derived pericytes and cord blood-derived MSCs partially prevented and rescued lung function and structure. However, the low cell engraftment suggested a paracrine effect. In line, cell free-derived CM from both cell types manifested significant benefit when used either prophylactically or therapeutically. Altogether, these studies open new avenues to exploit pericytes in neonatal regenerative medicine.

## 4 Evidence for paracrine healing properties of cardiac stem/progenitor cells

There is a growing consensus that the beneficial effects of any myocardial autologous or allogeneic cell therapy so far tested is, at least in part, mediated by a paracrine effect on the patient's cells at risk and activation of the host's CSCs by growth factors secreted by the transplanted cells (Braunwald, 2015, Lorkeers et al., 2014). Major contributors to this cardioprotective and CSC stimulatory effect are HGF and IGF-1 acting through their relative receptors present both on the myocytes, vascular and CSCs of the recipient's heart (Ellison et al., 2011).

MSCs have a broad repertoire of secreted trophic and immunomodulatory cytokines; however, they also secrete factors that negatively modulate cardiomyocyte apoptosis, inflammation, scar formation, and pathological remodeling (Ranganath et al., 2012). Moreover, it is questionable whether they are the most optimal cell to use regarding survival and homing to and engraftment in the myocardium. Furthermore, cells can become entrapped in the microvasculature and impede cell entry into the myocardium.

The safety and efficacy of transplanting 2 million allogeneic, mismatched Cardiosphere-derived cells (CDCs) was tested in infarcted rats. Three weeks post-MI, animals that received allogeneic CDCs exhibited smaller scar size, increased infarcted wall thickness, and attenuation of ventricular remodeling. Allogeneic CDC transplantation resulted in an improved fractional area change (∼ 12%), ejection fraction (∼ 20%), and fractional shortening (∼ 10%), and this was sustained for at least 6 months. Furthermore, allogeneic CDCs stimulated endogenous regenerative mechanisms (cardiomyocyte cycling, recruitment of endogenous CSCs, angiogenesis) and increased levels of myocardial VEGF, IGF-1, and HGF (Malliaras et al., 2014).

We have previously shown that CSCs that express high levels of the transcription factor GATA-4 exert a paracrine survival effect on cardiomyocytes through increased IGF-1.

secretion and induction of the IGF-1R signaling pathway (Kawaguchi et al., 2010). Furthermore, unlike bone marrow derived cells (Hofmann et al., 2005), CSCs have a very high tropism for the myocardium (Ellison et al., 2013). This cardiac tropism is governed by the SDF1-CXCR4 signaling axis, and when CSCs are injected either intracoronary or systemically, they home to and nest into the damaged heart with a high efficiency and significantly restore the myocardium, anatomically and functionally (Ellison et al., 2013).

**Fig. 3:**
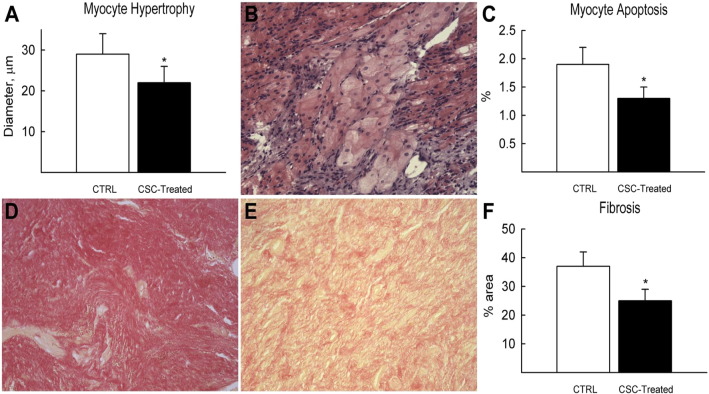
Through paracrine mechanisms, c-kit^pos^ heterologous HLA non-matched CSC treatment preserves myocardial wall structure and attenuates remodeling in a porcine MI model. (A) CSC treatment led to significantly decreased myocyte hypertrophy in the border region. *P < 0.05 vs. CTRL. (B) Representative H&E staining showing a band of hypertrophic myocytes in the border region of CTRL pig myocardium. (C) CSC treatment significantly decreased percent number of apoptotic (caspase 3 positive) myocytes in the border region. *P < 0.05 vs. CTRL. (D & E) Representative images of sirius red staining to identify fibrotic tissue (red) and muscle (yellow) in the infarct region of CTRL (D) and CSC-treated (E) pig hearts. (F) CSC-treated pig hearts had a decreased percentage area fraction of fibrosis in the infarct zone.*P < 0.05 vs. CTRL.

Cloned male eGFP-transduced heterologous HLA not-matched porcine CSCs, were administered intracoronary at differential doses (5 × 10^6^, 5 × 10^7^ and 1 × 10^8^) in 3 groups of pigs, 30 min after coronary reperfusion. Pig serum was injected to 6 control pigs after MI. BrdU was administered via osmotic pumps to track myocardial regeneration. Pigs were sacrificed at 30 min, 1 and 21 days. We found that heterologous CSC administration was well tolerated and without adverse effects. CSCs nested into the damaged myocardium with an efficiency of > 95%, at 30 min through to 1 day after MI. Minimal spill over of CSCs was detected in the coronary sinus, spleen, lung or live and all injected CSCs had disappeared from the myocardium at 21 days. CSC-treated infarcted pig hearts showed a significant increase in the number of endogenous c-kit^pos^ (GFP^neg^) CSCs in the border and infarct regions, compared to CTRL. CSC-treated hearts exhibited an increase in the number of small, newly formed BrdU^pos^ myocytes and capillaries. CSC injection significantly preserved myocardial wall structure and attenuated remodeling by reducing hypertrophy, apoptosis and fibrosis (Fig. 3). Moreover, cardiac function was significantly preserved/improved by heterologous CSC-treatment. To summarize, intracoronary injection of heterologous CSCs after MI in pigs, which is a clinically relevant MI model, activates the endogenous CSCs through a paracrine mechanism resulting in improved myocardial cell survival and physiologically meaningful regeneration (Ellison et al., 2009).

Allogeneic CSC therapy is conceptually and practically different from any presently in clinical use. The proposed cell therapy is only a different form of growth factor therapy, where the cells naturally home to the damaged myocardium, deliver a more complex mixture of growth factors, elicit a ‘paracrine’ effect and activate the endogenous target cells. Then the allogeneic CSCs are eliminated, and the regeneration triggered by activated endogenous CSCs is completely autologous. Therefore, the allogeneic CSCs survived long enough in the allogeneic host to produce their paracrine effect before being eliminated by the host immune system. Once more information is available, the allogeneic cells could be used either alone or in combination with the available factor therapy to improve the activation of the CSCs and the maturation of their progeny.

## 5 Conclusions

The market analysis predicts protein and peptide-based drugs will compose > 50% of novel drugs within the next 10 years. Pharmacological companies have been using unbiased discovery methods to generate new druggable compounds since many years. The approach is now increasingly used in academic research. It is likely that new cardiovascular drugs will be introduced in the next future by applying these approaches to the study of stem cell paracrine function. Due to the variability of the product and high costs associated with clinical grade production, stem cell-based therapy is not amenable to the majority of ischaemic patients. The use of drugs inspired by the stem cell secretome will instead offer unprecedented therapeutic opportunities, resulting in a fundamental shift in the initial concept of regenerative medicine.

## Conflict of interest

The authors do not have any conflict of interest to declare.
